# Viral Quasispecies Assembly via Maximal Clique Enumeration

**DOI:** 10.1371/journal.pcbi.1003515

**Published:** 2014-03-27

**Authors:** Armin Töpfer, Tobias Marschall, Rowena A. Bull, Fabio Luciani, Alexander Schönhuth, Niko Beerenwinkel

**Affiliations:** 1 Department of Biosystems Science and Engineering, ETH Zurich, Basel, Switzerland; 2 SIB Swiss Institute of Bioinformatics, Basel, Switzerland; 3 Centrum Wiskunde & Informatica, Amsterdam, The Netherlands; 4 Inflammation and Infection Research Centre, School of Medical Sciences, UNSW, Sydney, Australia; Heinrich Heine University, Germany

## Abstract

Virus populations can display high genetic diversity within individual hosts. The intra-host collection of viral haplotypes, called viral quasispecies, is an important determinant of virulence, pathogenesis, and treatment outcome. We present HaploClique, a computational approach to reconstruct the structure of a viral quasispecies from next-generation sequencing data as obtained from bulk sequencing of mixed virus samples. We develop a statistical model for paired-end reads accounting for mutations, insertions, and deletions. Using an iterative maximal clique enumeration approach, read pairs are assembled into haplotypes of increasing length, eventually enabling global haplotype assembly. The performance of our quasispecies assembly method is assessed on simulated data for varying population characteristics and sequencing technology parameters. Owing to its paired-end handling, HaploClique compares favorably to state-of-the-art haplotype inference methods. It can reconstruct error-free full-length haplotypes from low coverage samples and detect large insertions and deletions at low frequencies. We applied HaploClique to sequencing data derived from a clinical hepatitis C virus population of an infected patient and discovered a novel deletion of length 357±167 bp that was validated by two independent long-read sequencing experiments. HaploClique is available at https://github.com/armintoepfer/haploclique. A summary of this paper appears in the proceedings of the RECOMB 2014 conference, April 2-5.

## Author Summary

Humans infected with a virus, such as the human immunodeficiency virus (HIV-1) or hepatitis C virus (HCV), host a population of billions of virus particles. Among these, there is an unknown number of genetically different strains, some of which can harbor drug resistance and immune escape mutations. It is of clinical importance to know the DNA sequences and abundances of these variants, as they can affect treatment outcome. Here, we present HaploClique, a computational approach to reconstruct these sequences and to predict large insertions and deletions from paired-end next-generation sequencing data. Using simulations, we demonstrate that HaploClique can reconstruct full-length HIV-1 variants from low-coverage samples. Using real-world clinical data, we predict a novel deletion of 357±167 bp in a HCV patient sample that has been validated by two independent long-read sequencing experiments.

This article is associated with RECOMB 2014.

## Introduction

Genetic diversity is an important characteristic of evolving populations and it affects the chances of survival in changing environments. Assessing the genetic diversity of a population experimentally is generally labor-intensive and difficult. Populations of individual cells or viruses, however, can be analyzed efficiently using next-generation sequencing (NGS). Although single-cell approaches are still immature, direct NGS of mixed samples at deep coverage allows for probing populations in great detail. The challenges with this bulk sequencing approach are (i) to separate sequencing errors from genetic variation, (ii) to assemble the short NGS reads into an unknown number of different, unknown, longer haplotype sequences, and (iii) to estimate their frequency distribution.

Viruses such as human immunodeficiency virus (HIV-1) and hepatitis C virus (HCV) populate their hosts as swarms of related but genetically different mutant strains, each defined by its haplotype sequence. The structure of such a mutant cloud, which is often referred to as a viral quasispecies [1], is of clinical importance, because it has been shown to affect virulence [2] and pathogenesis [3]. In addition, low-frequency genetic variants may harbor resistance mutations that are capable of evolutionary escape from the selective pressure of host immune responses [4] and of medical interventions, such as anti-viral drug treatment [5]. NGS is currently introduced into clinical diagnostics, but the *de facto* standard procedure for assessing the quasispecies structure is simply based on single-nucleotide variant (SNV) calling. This approach allows only for estimating the per-site allele frequency spectrum of the virus population and it ignores patterns of co-occurrence among mutations. This limitation is critical, because epistatic interactions are abundant in RNA viruses [6]. Hence, one cannot predict viral phenotypes without knowing the underlying mix of haplotypes. Here, we address this challenge and present a computational approach for the viral quasispecies assembly problem.

The viral haplotype reconstruction problem is related to the human haplotype reconstruction problem, but it differs in several key aspects and faces different challenges. First, the number of unique haplotypes in a viral quasispecies is unknown unlike in the case of human diploid genomes. Second, viral populations typically exhibit more than two variants at each polymorphic locus and often all four different nucleotides. Hence, viral haplotypes cannot be described by binary sequences. Third, in a viral quasispecies, low-frequency variants are abundant and of clinical importance, yet they are difficult to distinguish from technical sequencing errors. Finally, RNA virus genomes are orders of magnitude shorter than the human genome, but exhibit more diversity within one host than the ∼0.1% diversity between the two parental human haplotypes [7].

Several methods for viral haplotype reconstruction have been developed in recent years, specialized for different NGS technologies, experimental designs, and quasispecies structures. In general, reconstruction can be performed either locally, in a genomic region that can be covered by the average read length, or globally, over longer regions such that overlapping reads are necessary for assembly. Local reconstruction means estimating the number of locally unique haplotype sequences and, at the same time, correcting sequencing errors. Probabilistic clustering [8]–[11] and k-mer statistics [12] have been proposed for this task. Global reconstruction is more challenging, as it requires computational solutions for assembling NGS reads, which has proven itself to be demanding even in settings without poly-ploidy [13].

For quasispecies assembly, approaches from different domains have been developed: (i) probabilistic mixture models [14], (ii) hidden Markov models [15], (iii) sampling schemes [16], (iv) combinatorial approaches based on analyzing the read overlap graph [8], [17]–[19], (v) coloring of overlap and conflict graphs by constraint programming [20], and (vi) exploiting the “identical by descent” information [21] in the HapCompass framework [22], originally designed for diploid single nucleotide polymorphism data.

The performance of global haplotype reconstruction depends on several factors, including the true underlying diversity of the population, the distribution of amplification and sequencing errors, the read length, and the distribution of the read coverage along the genome [23]–[25]. A major shortcoming of all existing methods is that they are unable to handle large insertions or deletions (indels). For example, large deletions can result from erroneous replication or, as observed recently in HIV-1, they may occur as alternative splice variants [26]. In the context of analyzing structural variation in the human genome, such as indels of varying sizes, the use of paired-end reads has been instrumental. For viral haplotype reconstruction, however, approaches that systematically exploit paired-end information are lacking.

In this paper, we present a new quasipecies assembly method for paired-end reads, called HaploClique, based on enumeration of maximal cliques (max-cliques) as a general approach to clustering NGS paired-end reads. Although, in general, the runtime of enumerating all max-cliques in a graph is exponential, it has recently been shown that the graphs induced by overlapping NGS reads can be handled efficiently [27], [28]. Here, we exploit this fact for the quasispecies assembly problem and develop a probabilistic model of sequence and structural similarity between reads.

Using max-clique enumeration for reference-based read assembly is orthogonal to combinatorial approaches for *de novo* assembly that rely on path finding in de Bruijn or similar graphs [29]–[31]. Instead of computing paths, we iteratively transform max-cliques into super-reads and then seek max-cliques of super-reads, thereby obtaining haplotype segments of increasing length. The haplotype segments can eventually be extended to global haplotypes if the degree of heterogeneity of the viral quasispecies is high enough. HaploClique is related to max-cut-driven approaches in human haplotype reconstruction [32], but the computational complexity of those approaches is prohibitive for virus populations of high and unknown ploidy. While HaploCliques enumerates all max-cliques, a max-cut approach seeks an optimal cut of the overlap graph.

HaploClique explicitly incorporates paired-end information for assembling viral haplotypes. We define the insert as the unsequenced fragment between the two ends of a paired-end read. We use linkage information among variant alleles in the distant pairs to identify reads that stem from the same haplotypes and generate error-corrected paired-end super-reads. Paired-end reads allow to bridge homogeneous, and hence ambiguous, genomic regions if the insert size is sufficiently large. They also increase the statistical power to distinguish local haplotypes from sequencing errors in homogeneous regions if the paired read is located in a more heterogeneous region. Employing our iterative clique enumeration procedure, we show that error-free full-length HIV-1 viral haplotypes can be reconstructed in a heterogeneous mix of five viral strains *in silico* from a data set with mean coverage of 600×. Furthermore, we demonstrate that, unlike existing methods, HaploClique can detect large indels in mixed virus populations *in silico* and *in vivo*. Finally, we apply HaploClique to a HCV Illumina paired-end NGS data set and predict a novel deletion of length 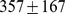 bp that has been confirmed independently by two long-read NGS platforms.

## Results

**Figure 1:**
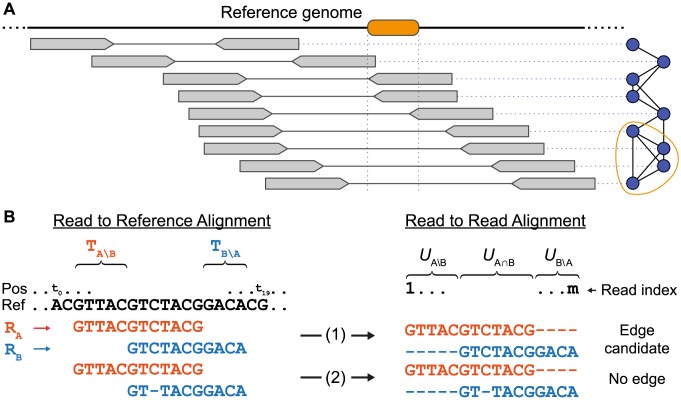
Max-clique enumeration and edge definitions. (A) Example of a read alignment graph based on the insert size criterion. Alignments of read pairs are shown in gray and the corresponding nodes in the graph representation are depicted in blue. The four bottom-most alignment pairs stem from a haplotype harboring a deletion (shown in orange in the reference genome) and therefore display a larger insert size than the remaining alignment pairs. Note that the four deletion-indicating alignment pairs form a max-clique (circled in orange). (B) Illustration of the compatible gaps condition of the sequence similarity criterion. Two reads 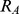 and 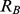 are aligned against the reference (left). This induces a direct read-to-read alignment of 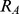 and 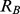 (right). Case (1): No gaps in the reference alignments lead to a gapless read-to-read alignment, which renders the pair of reads an edge candidate. Case (2): Gaps in the reference alignment lead to gaps in the read-to-read alignment, excluding the possibility of an edge. See also Figure S6 in the appendix for more complicated cases involving gaps.

We developed and implemented HaploClique, a computational viral quasispecies assembly method for paired-end NGS data. HaploClique defines a read alignment graph, in which each node corresponds to a single-end or paired-end alignment (Figure 1A). We draw edges between two nodes if the two corresponding alignments have sufficient overlap and are likely to stem from the same haplotype (Figure 1B). Each max-clique in this graph consists of a large number of reads from the presumed same haplotype segment. Thus, the consensus sequence of all reads in a max-clique is a prediction of a local haplotype sequence. We refer to such a consensus sequence as a super-read. This consensus sequence also serves to correct errors in the reads that participate in the super-read by replacing the sequence of the original reads with the consensus. This form of error correction benefits from phasing sequential variants through super-read construction. Paired-end reads are particularly helpful, as they allow to also phase distantly co-occurring variant alleles.

HaploClique proceeds by iterating (i) (super-)read alignment graph construction, (ii) max-clique enumeration, and (iii) super-read construction, until convergence. The lengths of the super-reads increase while iterating and convergence is established when super-reads have reached their maximum length. If the mixed sample is sufficiently heterogeneous, super-reads will eventually represent haplotypes of full length. Because we process paired-end reads and incorporate insert-size compatibility into our edge definition, we can also identify max-cliques that indicate larger insertions and deletions. These structural variations are recognized by too small or too large insert sizes among the alignments of the reads that participate in a max-clique. We analyzed HaploClique's performance on simulated data and demonstrate its use on *in vivo* HCV quasispecies sequencing data.

### Simulation studies

HaploClique integrates paired-end and base quality information for improved sequencing error correction and haplotype frequency estimation, which we assess first. Second, we evaluate HaploClique's behaviour when confronted with low heterogeneity among the different haplotype strains. Third, we demonstrate HaploClique's ability to detect large insertions and deletions in the quasispecies by making use of paired-end information. Fourth, we evaluate the quality of the local and global haplotypes that HaploClique predicts. Lastly, we compare HaploClique to state-of-the-art tools ShoRAH [33], PredictHaplo [14], and QuRe [16] in quasispecies reconstruction of a simulated five virus mix of well-known HIV-1 lab-strains.

In all of the following experiments, we simulated Illumina 2×250 bp paired-end reads using SimSeq [34] with fragment size 600 bp. To make the simulated data as realistic as possible, we estimated the required error profiles from an in-house MiSeq data set of a mixture of known HIV-1 strains. The average error-rate was 0.33% per base.

#### Error correction and frequency estimation

We assessed HaploClique's performance in error correction and frequency estimation, and their dependency on coverage and relative haplotype abundance. We generated ten HIV-1 strains by substituting ten percent of the nucleotides of strain 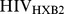. Positions to be substituted were sampled uniformly, separately for each of the ten strains. We sampled reads from these ten strains at coverage rates that resulted in abundance levels of 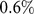, 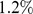, 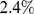, 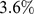, 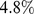, 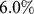, 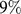, 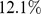, 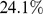, and 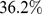.

**Figure 2:**
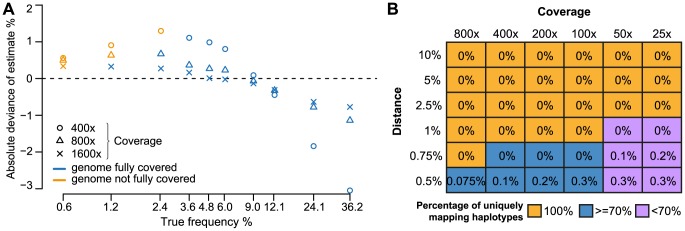
Performance in (A) frequency estimation and (B) distinguishing reconstructed local haplotypes. (A) Ten haplotypes were sampled with different frequencies (x-axis, logarithmic scale), and the mean deviations of the estimated to the true frequencies are reported for ten repetitions of the simulation (y-axis). The different symbols represent data sets with coverages 400×, 800×, and 1600×. Color indicates whether the genome was fully covered by predicted haplotypes (blue) or not (orange). (B) Performance in distinguishing reconstructed local haplotypes, depending on pairwise distance and coverage. The displayed percentages are the fractions of super-reads that do not match any true haplotype without error. Color-coded is the fraction of super-reads that match exactly one true haplotype (100%, orange; 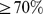, blue; 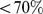, violet).

We measured the accuracy of estimating relative haplotype frequencies for different true abundances and coverages (Figure 2A). For each coverage, we repeated the simulation ten times and depict the mean deviances. For example, a mean deviance of +1% for a strain of frequency 4.8% translates to an estimate of 5.8%. The estimated frequencies approach the true ones for increasing coverage, as indicated by approaching the dashed line of 0% deviance. HaploClique tended to underestimate frequencies above and slightly overestimated frequencies below a true frequency of ten percent. For 1600× coverage, the absolute deviation was always below one percent. We additionally measures the frequency estimation robustness by computing the standard deviation for each true frequency and coverage (Figure S1). We observed increased robustness with increasing coverage, except for the cases where the super-reads do not fully cover the genome.

We assessed HaploClique's paired-end error correction capabilities using the same simulated data set. All super-reads, regardless of frequency, perfectly agreed with the respective true haplotype sequences. For frequency-coverage combinations of (3.6%, 400×), (2.4%, 800×), (1.2%, 1600×) and higher, the genomes were fully covered by super-reads (Figure 2A). For lower frequencies, reconstructed local haplotypes do not fully cover the genome in all ten data sets.

#### Minimal variant heterogeneity

To investigate the minimal heterogeneity necessary to distinguish between different haplotypes, we simulated datasets of two different haplotypes, varying coverage rates between 25 and 800× and pairwise distance from 0.05% to 10%.

We measured the false positive rates, percentages of reconstructed haplotypes with at least one false nucleotide, and percentages of perfectly reconstructed haplotypes that map uniquely to exactly one true variant (Figure 2B). For distances of 1% and above, reconstruction was perfect at all coverage rates. For distances of 0.75% and below, the vast majority of the reconstructed haplotypes still perfectly match a true haplotype. In general, the false positive rate decreases with higher coverage. However, with decreased distance between true haplotypes, many regions are conserved and cannot be assigned uniquely to a single true haplotype.

#### Large deletion prediction

The edge definition of HaploClique's read alignment graph allows for identifying large indels in the haplotypes (Methods). Thanks to the insert size criterion, one can predict indels from paired-end read information despite the lack of alignments of read ends that directly cover the deletion breakpoint. Such alignments may be lacking in practice, because even the most advanced read mappers have difficulties aligning reads across long indels.

**Figure 3:**
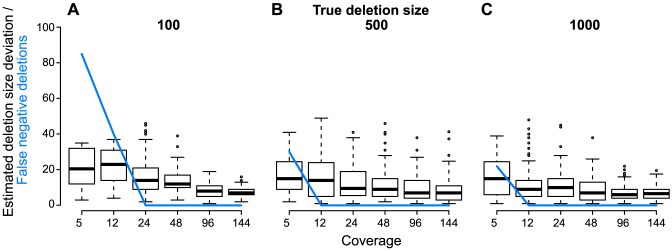
Large deletion estimates. Estimated deletion size deviation and false negative rate for different true deletion sizes of (A) 100, (B) 500, and (C) 1000 bp. For each deletion length and each coverage of 5, 12, 24, 48, 96, and 144×, a boxplot summarizes the deviations of the estimated to the true deletion size in 100 simulated samples. The blue line represents the number of false negative predicted deletions in each of the 100 samples.

In this simulation, we benchmarked the false negative rate of the predicted deletions and the deviations of the estimated to the true deletion lengths (Figure 3). Aiming at a simple, yet instructive benchmark data set, we created a new haplotype by randomly placing three deletions of sizes 100, 500, and 1000 bp into the 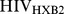 genome. We simulated reads from the reference haplotype with a mean coverage of 100× and from the deletion-harboring haplotype with coverages of 5, 12, 24, 48, 96, and 144×. We sampled 100 data sets for each coverage to account for variability in the sampling process. Comparing coverages of 5 and 144×, we observed a more reliable size estimate with increasing coverage, as the standard deviation decreased by a factor of up to two. Independent of the true deletion size, the estimated median size approached the true size up to 6 bp. For the true deletion sizes of 100, 500, and 1000 bp the number of false negatives decreased to zero for a coverage of 24, 12, and 12×, respectively. The median size deviation to the true deletion size was always below the standard deviation of the insert fragment size if there was sufficient coverage, i.e., no false negatives.

#### Global haplotype assembly

We benchmarked HaploClique's performance in terms of genome coverage, as well as numbers and lengths of constructed super-reads. We simulated a heterogeneous population, henceforth referred to as *lab-mix*, of five lab strains, namely 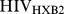, 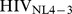, 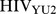, 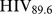, and 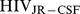. Among these strains, pairwise distances vary per gene and along the entire genome between 1 and 16% and between 2 and 6%, respectively. We sampled reads uniformly with a mean coverage of 600×, i.e., 120× per strain. This low coverage corresponds to very low-frequency variants in datasets of higher coverage (commonly 10,000 to 100,000×), which are of interest in diagnostic applications.

**Figure 4:**
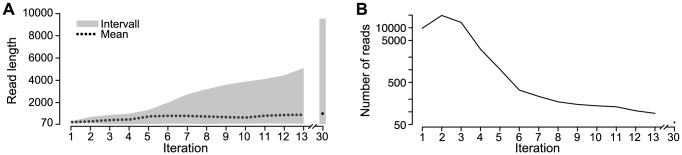
Global haplotype assembly results. Minimum, maximum, and mean read lengths (A) and the total number of reads (B) for the global haplotype assembly of the lab-mix, for the first 13 and the last iteration (30).

HaploClique was able to reconstruct haplotype 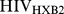 at its full length, without a single error (Table 1). The haplotypes of the other four variants grew up to a maximal size of 5-6 kb for the longest super-reads, where full length of the five true strains varied between 9 and 10 kb. For all strains, the reconstructed haplotypes covered the genome at its full length. The false positive rate (the rate of not perfectly matching super-reads) was 0.3%. The maximal read length increased monotonically for each iteration from 250 bp up to full-length of ∼10 kb (Figure 4). During the first two iterations, the number of super-reads increased, but from the third iteration decreased, converging to a number of 56 super-reads (Figure 4).

**Table 1:** Global haplotype assembly comparison.

	Estimated HIV-1 strain frequency (max. rel. haplotype length)							
Method	HXB2	NL4-3	YU2	89.6	JR-CSF	Error rate	# haplotypes	Precision
HaploClique	22% (100%)	21% (61%)	19% (59%)	18% (61%)	20% (57%)	0.012%	56	99.7%
ShoRAH	29% (97%)	20% (97%)	20% (97%)	9% (97%)	20% (99%)	1.99%	196	0%
PredictHaplo	91% (99%)	0% (0%)	1% (3%)	6% (12%)	2% (18%)	1.65%	95	0%
QuRe	46% (91%)	7% (91%)	0% (0%)	27% (91%)	20% (93%)	2.95%	15	0%

Global haplotype assembly comparison of HaploClique with the software packages ShoRAH [33], PredictHaplo [14], and QuRe [16]. We report the estimated variant frequencies and, in parenthesis, the maximal length of the reconstructed haplotypes relative to the genome length, for each of the five variants. In the remaining columns, the average error rate (computed as the number of mistaken nucleotides, divided by the length of the haplotype computed), the total number of reconstructed haplotypes, and the precision (percentage of perfectly reconstructed haplotypes weighted by the respective estimated frequency) are reported. See Methods for more details on frequency estimation.

#### Comparisons

We performed haplotype reconstruction for the lab-mix using the tools ShoRAH [33], PredictHaplo [14], and QuRe [16], and compared the results to those of HaploClique (Table 1). Hapler v1.60 [19] did not accept NGS alignments with insertions, and hence was not applicable to this heterogeneous virus populations. PredictHaplo v0.4 with paired-end option reconstructed local or global haplotypes with an average error-rate of 1.65%. ShoRAH does not take paired-end information into account and reconstructed 196 full-length genomes, 16 over 1% estimated frequency, with an average error rate of 1.99%. QuRe v0.99971 performed its own single-end alignment and reconstructed global haplotypes with an average error rate of 2.95%, the highest among all methods. The wall-clock time and maximal memory consumption, respectively, was 10 min and 12 mb for PredictHaplo, 30 min and 150 mb for HaploClique, 1.5 hr and 680 mb for ShoRAH, and 2 hr and 33 gb for QuRe on a 80-core server with 1 tb memory. PredictHaplo and HaploClique used a single thread, QuRe and ShoRAH are partially multithreaded. The results of QuRe were not reproducible, because new instances of QuRe on the same data set did not finish within one day. HaploClique performed best in maximal reconstructed haplotype length that was error-free, and it had the closest estimated haplotype distribution and overall the highest precision.

### Patient sample

For the application of HaploClique to a clinical sample, HCV RNA was extracted from the plasma collected from a subject isolated 135 days post infection and the NS5 region RT-PCR amplified as previously described [35]. In this subject there was experimental evidence of antigen-specific CD8+ T cell responses targeting two epitopes in the NS5 region (K2629SKRTPMGF and W2820LGNIIMFA). The NS5B region encodes for the RNA-dependent RNA polymerase and is essential for the replication of the virus.

This amplicon was sequenced to a coverage of 80,000× on a MiSeq instrument using a 2×250 bp read kit. The resulting reads were aligned using BWA-MEM [36]. We found the insert size distribution to have a mean of 155 bp and a standard deviation of 167 as estimated by HaploClique. Despite this large standard deviation, HaploClique was able to discover a 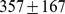 bp deletion. No other indels were reported by HaploClique.

In two independent sequencing runs of the same amplicon, once on a 454/Roche GS FLX+ system and once on a PacBio instrument, the presence of the deletion was confirmed. Both technologies yield longer reads than MiSeq that could successfully be aligned across the deletion breakpoint, allowing to determine breakpoint coordinates at base-pair resolution.

For the alignment of the longer reads, extreme affine gap costs have been used to find the deletion. In general, this leads to alignment artifacts in other regions, causing false positive haplotype calls. With a read length of 250 bp, we did not succeed to align reads across the large deletion.

Comparing coordinates, we found that the start positions predicted by HaploClique was 15 bp off the true position and the true length amounted to 444 bp. That is, the length difference between true and predicted deletion amounted to 87 bp, or 0.52 standard deviations.

## Discussion

We have presented HaploClique, a method for local haplotype reconstruction, structural variant detection of large insertions and deletions, and global haplotype assembly, which represents a principled approach to viral quasispecies assembly from NGS paired-end reads. HaploClique builds on a read alignment graph as underlying combinatorial model, where nodes correspond to single-end or paired-end alignments of reads. Edges are modeled in a probabilistic fashion.

They are based on sequence similarity of the read overlap by incorporating phred-style quality scores in combination with a position-wise prior for the non-overlapping parts of the reads, and on a criterion that measures insert size compatibility of the two alignments. While the sequence similarity criterion accounts for correct assembly of reads, the insert size criterion allows for detecting insertions and deletions in viral haplotypes that cannot be detected from single-end read alignments alone. We suggest a model that unifies sequencing error correction, clustering reads into haplotype groups, as well as assembling reads into longer fragments, all of which naturally emerge from the model.

In the read alignment graph, max-cliques represent maximal read sets that overlap and represent (locally) identical haplotype sequence. The advantage of the max-clique computation is twofold. First, it clusters reads, thereby separating reads stemming from different haplotypes.

Second, it enables sequencing error correction in a way that can make full use of co-occurrence, that is, statistical correlation of variant alleles within reach of the reads participating in a max-clique. In particular, the error correction exploits paired-end information if provided. The improved error correction is important, as it gives rise to improved frequency estimates and allows for distinguishing between haplotypes whose pairwise distance is below 1%.

HaploClique allows for reconstructing full-length global haplotypes using a read assembly procedure that is orthogonal to all existing assembly methods. In our iterative approach, we alternate between transforming max-cliques into super-reads, which form the nodes of a new alignment graph, and finding max-cliques in the new graph. We repeat this process until convergence, which is established when super-reads do not grow any longer.

HaploClique depends on three parameters to be adjusted manually: minimal read overlap, 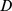, a threshold for the probability that two overlapping reads stem from locally identical haplotypes, 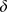, and the minimal coverage to call the super-read sequence 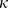. In general, if one of the parameters is decreased, the number and size of cliques will increase. If 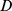 and 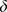 are too small, the purity of cliques will decrease, meaning reads from different but very similar haplotypes cluster. If 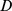 is too large, cliques will grow slower and less frequent haplotypes may be missed. If 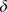 is too large, reads are more likely to cluster not only if they stem from the same haplotype but also if they have technical errors in common; this leads to lower error correction efficiency. If 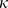 is too small, there might not be enough statistical power to correct for sequencing errors. If 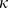 is too large, the false negative rate will rise, as low-frequency haplotypes do not provide enough reads to form cliques. We used two different parameter sets for HaploClique. In the first iteration, local haplotype reconstruction with error correction is performed and we chose 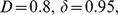 and 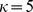. In practice, the results are insensitive to the parameter choice (Figure S2). For the following iterations, the quasispecies assembly, we assume that haplotypes are error-corrected and must match perfectly. We set 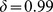 to account only for the stochasticity of the Phred scores.

We evaluated HaploClique by extensive simulation studies. The simulated haplotypes were well-known and much analyzed HIV-1 virus strains. We kept coverage in the simulation study rather low, so as to evaluate our tool in the presence of only weak signals. We did this also in comparison with extant state-of-the-art tools. We demonstrated that our approach has superior error correction capabilities. This, in turn, yields accurate haplotype frequency estimates, even at the rather low coverage of 120× per haplotype. The tools we compared to were not able to provide similarly accurate frequency estimates. HaploClique proved to be insensitive to a coverage reduction of one order of magnitude with respect to prevalent sequencing experiments, which commonly operate at 5000× or higher.

Beyond improved frequency estimates, we also improve haplotype sequence reconstruction. In all experiments, more than 99% of the haplotype segments we predict perfectly matched true haplotype sequences. None of the other tools generated even only one such perfectly matching segment, possibly because they require much higher coverage. This improvement in terms of accuracy may be due to the probabilistic model that treats error correction and assembly within one unifying framework. Our simulations also indicated that the degree of heterogeneity required in order to reconstruct large enough haplotype segments can be lower than 1%.

We also ran HaploClique on a real, Illumina MiSeq dataset of coverage 80,000×, which was found to consist of two HCV strains one of which had a frequency of only approximately 3% and contained a deletion of size 444 bp, as conformed by independent 454/Roche and PacBio sequencing experiments. In the MiSeq dataset, the deletion in question could not be detected by state-of-the-art read alignment tools. HaploClique successfully predicts this deletion, despite the large standard deviation of the fragment size distribution (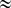 167 bp). These experiments document that our method can detect large deletions also in Illumina paired-end datasets that otherwise would be difficult to identify.

Despite these improvements over previous methods, there are limitations of this approach. For example, the runtime of HaploClique is exponential in the read coverage. This feature is critical in the first two iterations of the procedure, before the number of reads is decreased. We observed that, approximately, the runtime doubles for each additional 250 reads of coverage. The baseline runtime was ∼4 minutes for a data set with coverage 1000×, on a single 3 GHz core. To overcome this computational bottleneck, one may perform the first iterations of haplotype reconstruction on subsets of the data and then assemble the merged results. Another extension that may decrease the runtime is to employ improved clustering techniques [37].

In the future, we also plan to explore on human whole-genome data, including polyploid cancer genomes, to perform error correction of the paired-end reads by local haplotype reconstruction and to assemble diploid haplotypes. This problem is more challenging due to the larger genome size and smaller levels of diversity, but several ideas presented here and implemented in HaploClique may prove useful for this task.

## Methods

HaploClique performs paired-end error correction, local and global haplotype reconstruction, and structural variant calling based on enumerating cliques in the read alignment graph. We first explain the graph construction and then how super-reads are built, how global haplotypes are iteratively constructed, and how haplotype abundancies are estimated.

### Read alignment graph

Let 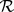 be the set of all reads from a viral quasispecies sequencing experiment and 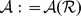 the set of their alignments to a reference genome as computed by a read aligner. In this paper, we assume that each read can be uniquely mapped, which is a reasonable assumption for short, non-repetitive viral genomes. In our experiments, we use the Illumina MiSeq technology for sequencing and BWA-MEM [36] as a read aligner. However, HaploClique depends on the sequencing technology and read mapper only insofar that it expects reads to be equipped with quality scores and that the reads can be properly aligned.

We construct a graph 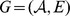 where the read alignments 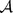 are the vertices. An edge 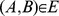 indicates that the two alignments 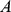 and 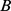 overlap sufficiently and that the corresponding reads are likely to originate from (locally) identical haplotypes. More precisely, we draw an edge between 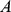 and 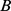 if they satisfy two criteria based on sequence similarity and insert sizes, respectively. While the sequence-based criterion ensures that the reads 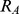 and 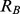 do not exhibit mutually contradictory sequences, the insert size-based criterion guarantees that 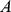 and 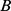 do not contradict each other in terms of their fragment sizes. We allow alignments 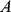 and 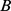 to be any combination of single- and paired-end reads, but the size-based criterion applies only if both alignment are based on paired-end reads.

#### Sequence similarity criterion

We define pairwise sequence distance as Hamming distance. We assume if reads stem from the same haplotype, their sequences are identical up to sequencing errors in the intervals of overlapping alignments.

The reference alignments 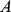 and 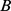 induce a direct read-to-read alignment of 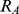 and 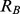. Let 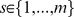 index the positions of the resulting read-to-read alignment (Figure 1). We consider the subset of positions 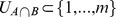 that are covered by both reads 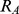 and 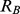. For 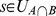, let 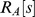 and 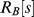 be the corresponding nucleotides or gap symbols (“
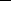
”). We construct the induced read-to-read alignment, so that no column contains gap symbols in both rows. If there is a gap 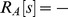 or 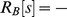 for some 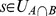, then we consider 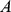 and 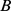 *incompatible* and do not connect them by an edge.

In the following, we assume a gapless read-to-read alignment, 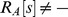 and 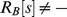 for all 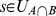. Each nucleotide in each read comes with a base calling quality score (phred score) determined by the sequencer. Let 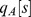 and 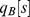 be the corresponding probabilities that 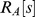 and 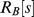, respectively, was sequenced erroneously. For 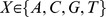, we define
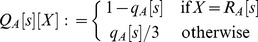
(1)and compute
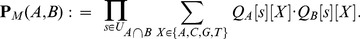
(2)



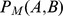
 is the probability that the underlying DNA sequences of 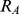 and 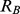 are identical on the overlap 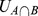. The actual reads 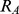 and 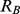 might differ on 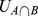 due to sequencing errors.

Let 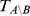 denote all reference positions covered by 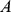 but not by 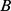. Let 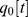 be the probability that the nucleotides of two randomly drawn reads coincide when being aligned with reference position t. This quantity can be estimated based on the empirical allele frequency distribution at position 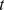, denoted 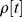. If 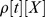 denotes the probability of observing nucleotide 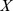 at position 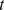, then 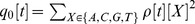. The probability that two randomly drawn alignments that, in contrast to 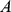 and 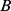, both cover 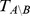 and 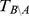, exhibit identical nucleotides at all these positions is
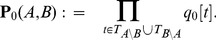
(3)


To finally decide whether two reads 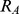 and 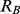 are likely to originate from (locally) identical haplotypes, we consider
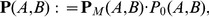
(4)and say that two alignments 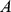 and 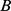 satisfy the *sequence similarity criterion* if

1. they do not contain incompatible gaps: 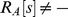 and 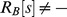 for all 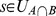,
2. there is sufficient overlap: 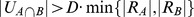, and
3. the probability that the two reads were sampled from the same haplotype is sufficiently large: 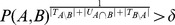, where the exponent ensures proper length normalization.

#### Insert size criterion

If a deletion or insertion is present, the distance between the reference alignments of the two ends increases (for deletions) or decreases (for insertions) compared to the situation without indels. Such insert size discrepancies can thus indicate the presence of indels as well as the (in)compatibility of two read pairs. Following [27], we define an insert size criterion based on this observation. This criterion only applies to aligments 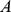 and 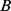 of two paired-end reads 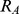 and 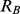.

Let 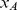 and 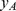 be the rightmost position of the left end and the leftmost position of the right end, respectively, of alignment 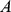. Let 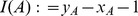 be the alignment interval length. For two overlapping alignments 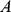 and 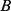, let 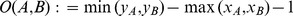 be the length of the overlap of the alignment intervals and 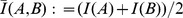 the mean interval length (Figure S3). Let 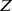 be Norm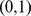-distributed and 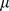 and 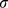 be mean and variance, respectively, of the empirical insert size distribution. The *insert* criterion is satisfied if
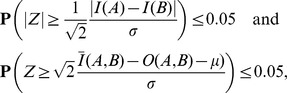
that is, if the alignments have similar interval lengths and sufficient overlap [27] (Figure S4).

#### Edge definition

HaploClique has two modes. When the input consists solely of paired-end reads with a known insert size distribution, then both the sequence similarity criterion and the insert size criterion can be used, and an edge is drawn if both criteria are satisfied. If this is not the case, only the sequence similarity criterion applies. In the former mode, the statistical power to distingiush different haplotypes is larger due to employing two independent criteria. This mode also allows for calling indels based on the average insert size of alignments in a clique. Applying only the sequence similarity criterion, on the other hand, comes with the flexibility of mixing paired-end reads, single-end reads, and super-reads.

### Max-clique enumeration

Cliques 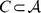 are fully connected subgraphs of the read alignment graph 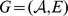. They indicate groups of reads that are all likely to stem from locally identical haplotypes. Hence max-cliques form maximal groups of reads that originate from locally identical haplotypes.

The algorithm proceeds by first sorting all nodes 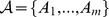 from left to right, in ascending order of the alignment coordinates such that 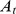 starts left of 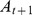. The algorithm then computes maximal cliques by processing all nodes 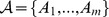 in this order. Let 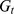 be the induced subgraph of 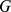 with vertices 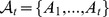 and let 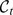 be all max-cliques in 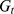. For a node 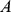, let 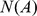 be all nodes that are connected to 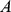 by an edge 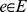. If the rightmost coordinates of all alignments in a clique 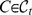 are smaller than the leftmost coordinate of 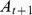, this clique cannot be further affected by any node 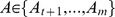 — such cliques are maximal in 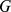 and can be output if only nodes 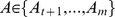 are left to be considered. After having processed all nodes 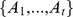, we declare all cliques 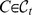 that can still be affected by nodes 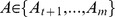 to be *active*.

When processing node 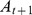, we compute its neighborhood 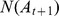 and add a new clique 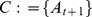 if intersecting 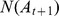 with each active clique yields the empty set. Otherwise, for each active clique 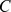, we set 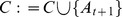 if 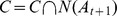, and we add a new clique 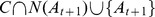 if 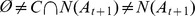. Among all new cliques to be added, we eliminate duplicates.

Max-clique enumeration is related to the problem of finding a minimum clique cover [38], where a minimal set of non-overlapping max-cliques is sought, whose vertices cover the graph.

#### Runtime analysis

Let 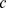 be an upper bound on the number of active cliques and 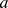 be an upper bound on the number of active nodes, where we call a node active if it is part of at least one active clique. Since no two distinct active cliques can be extended to the same max-clique, we have 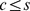, where 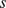 is the number of max-cliques in 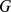. Let 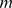 be the number of alignments 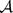. First, sorting all nodes requires 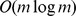 time. Processing each node requires 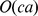 time (when doing the duplicate removal by radix sorting bit vectors representing the new cliques). Therefore, the total runtime is 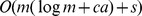. Since 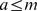 and 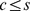, the algorithm has *output polynomial* runtime, that is, it is polynomial in the size of the input plus the size of the output.

In general graphs and in the worst case, the number of max-cliques is exponential in the number of nodes [39]. Thus, our algorithm is not necessarily polynomial in the input alone. In interval graphs, however, the number of cliques grows at most linearly and simple sweep algorithms can enumerate all max-cliques in linear time. In these graphs, each vertex is an interval on the real line and an edge is drawn between two vertices whenever the corresponding intervals overlap. The read alignment graph as defined above can be regarded as an interval graph with removed edges. Indeed, that two alignments overlap is necessary but not sufficient for drawing an edge. Although, formally, any graph can be constructed by removing edges from an interval graph, being close to an interval graph with bounded coverage is a property that one can exploit. Due to the “banded” shape of the graph (Figure 1), the number of maximal cliques grows linearly in the genome length (and thus in the number of nodes) in practice (Figure S5B). Under this assumption, the runtime of the clique finder we use [27] is also linear in the number of nodes once they have been sorted. This linear behavior was indeed observed in computational experiments using viral genome lengths differing by several orders of magnitude (Figure S5). We optimized the implementation of the algorithm by exploiting bit-parallelism whenever possible. Being linear in the genome length, in practice, is crucial for analyzing the haplotype structure of longer viral or bacterial, or eventually also cancer genomes. It is for this reason, that we have selected this algorithm and prefer it over fast general purpose algorithms for max-clique enumeration in sparse graphs [40], [41], which generally scale quadratically with genome length.

### Super-read assembly

Each max-clique is a set of reads with mutually compatible alignments. Therefore, we can construct consensus sequences for max-cliques, which we refer to as super-reads. The purpose of super-read construction is two-fold. First, super-reads represent haplotype segments. Second, super-reads can be used as input to further iterations of max-clique enumeration, with the goal of global haplotype reconstruction, which is discussed in the next section.

To construct super-reads, let 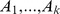 be the 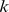 alignments participating in a max-clique and let 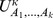 be the set of positions where at least 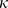 of these alignments contain non-gap characters. We recall that 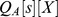 denotes the probability that nucleotide 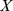 gave rise to position 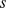 in read 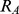, although 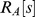 might differ from 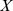 due to sequencing errors.

We determine the nucleotide sequence of the super-read 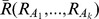 by means of a weighted position-wise majority vote. We set 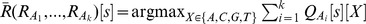, for each position 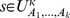, where 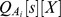 is defined to be zero when 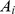 does not cover position 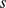. The parameter 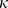 ensures that the super-reads have sufficient coverage of high quality. For later frequency estimation, we keep track of which original reads gave rise to which super-read.

### Global assembly strategy

For global haplotype reconstruction, or quasispecies assembly, we iterate the clique enumeration procedure. We align reads against the reference sequence, construct the read alignment graph, find max-cliques, and merge them into larger super-reads with updated phred scores that reflect corrected error profiles. In the next iteration, we use these super-reads as reads and restart the procedure until number and length of super-reads have converged. For the assembly step, we assume that reads have already been error-corrected in the first iteration and set 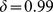. Reads have to match perfectly and 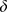 only allows for stochasticity of the Phred scores. We start the iterations with a relative overlap of 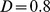. Once the length and number of super-reads converged, we decrease 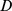 by 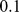 down to a minimum of 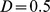.

### Haplotype abundance estimation

We estimate haplotype abundance by counting the number of (original) reads that participate in the super-reads giving rise to the haplotypes. Original reads may participate in several super-reads and thereby contribute to abundance counts for several haplotypes. We resolve this issue by keeping track of the original read in each iteration, such that each read can be assigned to the final haplotypes after convergence. Reads contributing to several haplotypes abundances are then taken into account by weighting them accordingly.

### Data

The MiSeq raw read data set is available through the Sequence Read Archive under the BioProject accession number SRP034655. The MiSeq 2×250 bp error profiles for SimSeq [34] used in the simulations are available at https://github.com/armintoepfer/haploclique under data.

A summary of this paper appears in the proceedings of the RECOMB 2014 conference, April 2-5 [42].

## Supporting Information

Figure S1 **Standard deviation of ten haplotype frequency estimates for different coverages.** Ten haplotypes were sampled with different frequencies (x-axis, logarithmic scale), and the standard deviations of the frequency estimates are reported for ten repetitions of the simulation (y-axis, logarithmic scale). The different symbols represent data sets with coverages of 400×, 800×, and 1600×. Color indicates whether the genome was fully covered by predicted haplotypes (blue) or not (orange).
(PDF)

Figure S2
**Empirical studies for parameters** 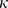 **and** 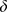 **.** Using the lab-mix, as described in the Results section, we benchmarked the reconstruction performance, with respect to error rate (left) and mean read length (right), for varying levels of 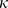, the number of reads required for initial super-read construction. Only sequence fragments that are supported by at least 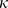 original reads within one max-clique are turned into super-reads of the first generation (iteration). Performance depends on the parameters 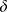, the threshold for the probability that both reads stem from the same haplotype and the minimal coverage 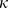 to create a consensus sequence of the super-read. We varied 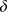 between 0.91 and 0.99, and 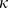 between 1 and 9.(PDF)

Figure S3
**Overlapping inserts.** Two alignment pairs 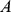 and 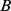 along with their insert sizes 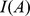 and 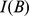 and their overlap 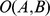 are shown.(PDF)

Figure S4 **Insert edge definitions.** The different scenarios (A)–(D) of the insert size criterion are shown.
(PDF)

Figure S5
**Runtime analysis.** (A) Run time and (B) number of max-cliques for varying genome lengths between 10 kb and 1 Mb and for coverages of 250× and 500×. Dots represent observed runtime in seconds (A) and number of max-cliques in the corresponding alignment graph (B). Lines represent linear regressions after log-log transformation. The slopes of approximately one indicate linear relationships. 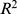 is the fraction of variance explained by the log-log linear model. For each of the five viruses HIV-1, PhiCh1, enterobacteria phage P1, Bacillus phage G, and Acanthamoeba polyphage moumouvirus, we simulated three haplotypes with a distance of five percent to the reference genome. For each virus, we generated data sets with mean coverage 250× and 500×.(PDF)

Figure S6
**Edge definitions.** Illustration of the compatible gaps condition of the sequence similarity criterion. Two reads 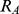 and 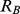 are aligned against the reference (left). This induces a direct read-to-read alignment of 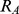 and 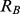 (right). Case (1): No gaps in the reference alignments lead to a gapless read-to-read alignment, which renders the pair of reads an edge candidate. Case (2): Gaps in the reference alignment become eliminated in the direct read-to-read alignment implying an edge candidate. Case (3): The reference alignment leads to aligning –‘C’ against ‘C-’, which we interpret as aligning C against C, that is, virtually case (2) is in effect. Case (4): Gaps in the reference alignment that lead to gaps in the read-to-read alignment exclude the possibility of edges. Case (5): Similar to (3), but we interpret ‘-A’ against ‘C-’ as gap implying that no edge is possible.(PDF)
