# Supplementary figures and images for: Viral Quasispecies Assembly via Maximal Clique Enumeration
Source: PLoS Comput Biol. 2014 Mar 27;10(3):e1003515. doi: 10.1371/journal.pcbi.1003515 (PMC3967922; doi:10.1371/journal.pcbi.1003515)

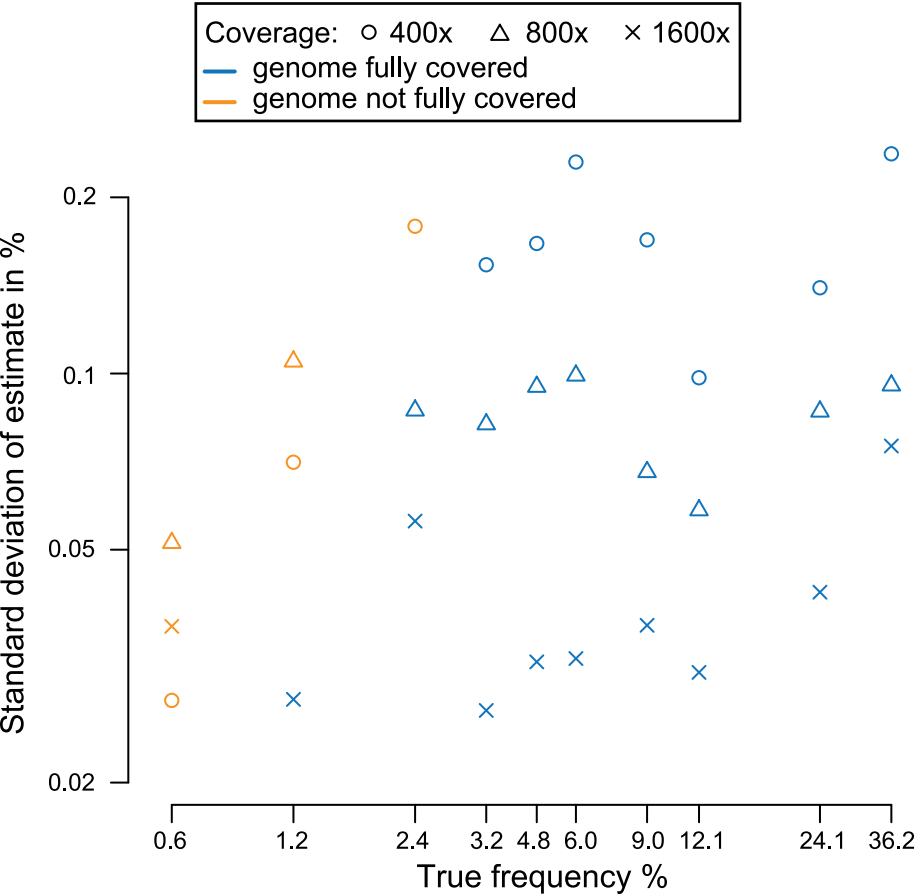

Supplement: Figure S1 — Standard deviation of ten haplotype frequency estimates for different coverages. Ten haplotypes were sampled with different frequencies (x-axis, logarithmic scale), and the standard deviations of the frequency estimates are reported for ten repetitions of the simulation (y-axis, logarithmic scale). The different symbols represent data sets with coverages of 400×, 800×, and 1600×. Color indicates whether the genome was fully covered by predicted haplotypes (blue) or not (orange). (PDF) [file pcbi.1003515.s001.pdf]

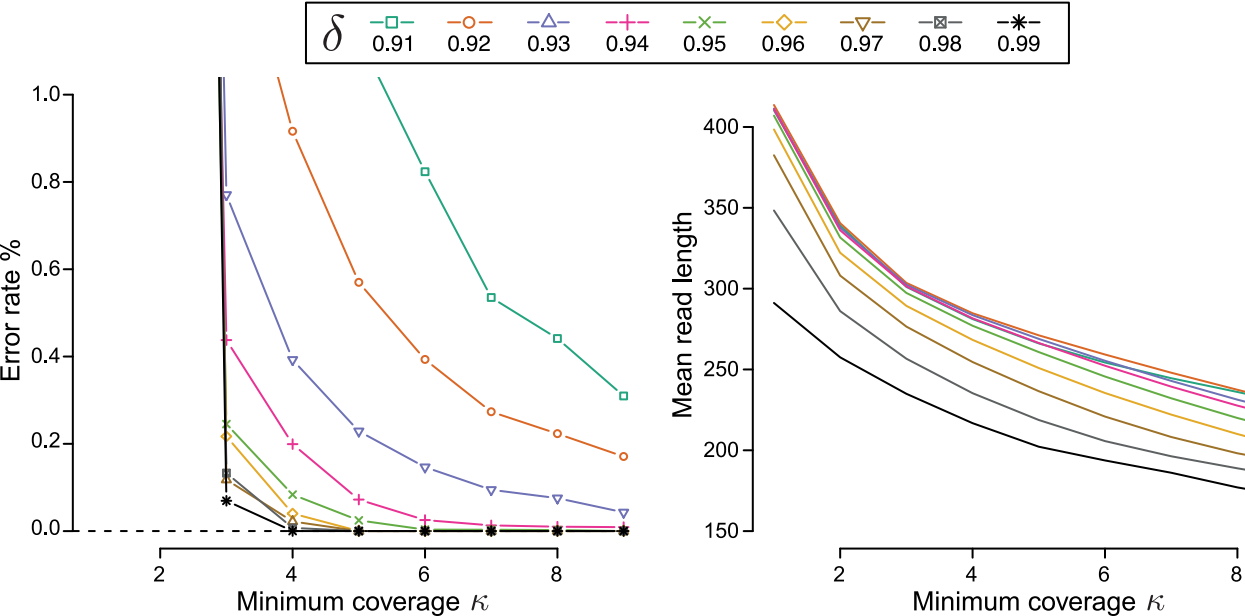

Supplement: Figure S2 — Empirical studies for parameters and . Using the lab-mix, as described in the Results section, we benchmarked the reconstruction performance, with respect to error rate (left) and mean read length (right), for varying levels of , the number of reads required for initial super-read construction. Only sequence fragments that are supported by at least original reads within one max-clique are turned into super-reads of the first generation (iteration). Performance depends on the parameters , the threshold for the probability that both reads stem from the same haplotype and the minimal coverage to create a consensus sequence of the super-read. We varied between 0.91 and 0.99, and between 1 and 9. (PDF) [file pcbi.1003515.s002.pdf]

# Reference genome

---

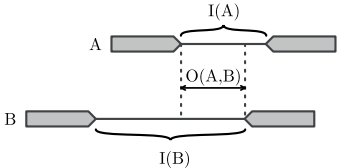

Supplement: Figure S3 — Overlapping inserts. Two alignment pairs and along with their insert sizes and and their overlap are shown. (PDF) [file pcbi.1003515.s003.pdf]
