# Supplementary material for: Viral Quasispecies Assembly via Maximal Clique Enumeration
Source: PLoS Comput Biol. 2014 Mar 27;10(3):e1003515. doi: 10.1371/journal.pcbi.1003515 (PMC3967922; doi:10.1371/journal.pcbi.1003515)
Supplement: Figure S4 — Insert edge definitions. The different scenarios (A)–(D) of the insert size criterion are shown. (PDF) [file pcbi.1003515.s004.pdf]

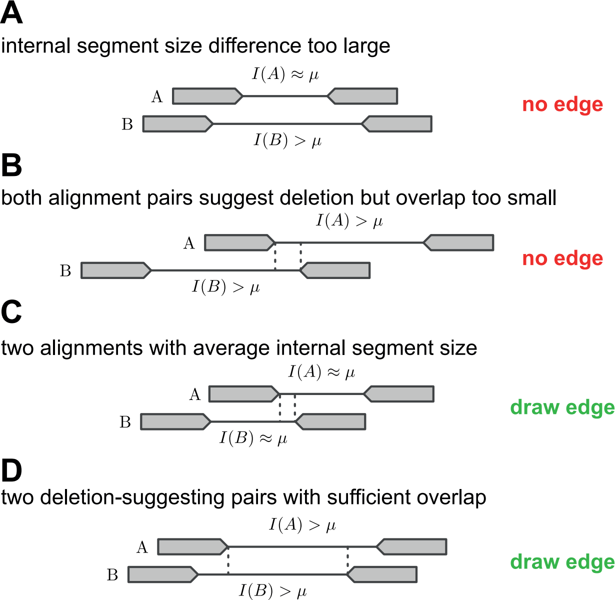
A
internal segment size difference too large
I(A) ≈ μ
A
B
I(B) > μ
no edge
B
both alignment pairs suggest deletion but overlap too small
I(A) > μ
A
B
I(B) > μ
no edge
C
two alignments with average internal segment size
I(A) ≈ μ
A
B
I(B) ≈ μ
draw edge
D
two deletion-suggesting pairs with sufficient overlap
I(A) > μ
A
B
I(B) > μ
draw edge
