# Supplementary material for: Viral Quasispecies Assembly via Maximal Clique Enumeration
Source: PLoS Comput Biol. 2014 Mar 27;10(3):e1003515. doi: 10.1371/journal.pcbi.1003515 (PMC3967922; doi:10.1371/journal.pcbi.1003515)
Supplement: Figure S5 — Runtime analysis. (A) Run time and (B) number of max-cliques for varying genome lengths between 10 kb and 1 Mb and for coverages of 250× and 500×. Dots represent observed runtime in seconds (A) and number of max-cliques in the corresponding alignment graph (B). Lines represent linear regressions after log-log transformation. The slopes of approximately one indicate linear relationships. is the fraction of variance explained by the log-log linear model. For each of the five viruses HIV-1, PhiCh1, enterobacteria phage P1, Bacillus phage G, and Acanthamoeba polyphage moumouvirus, we simulated three haplotypes with a distance of five percent to the reference genome. For each virus, we generated data sets with mean coverage 250× and 500×. (PDF) [file pcbi.1003515.s005.pdf]

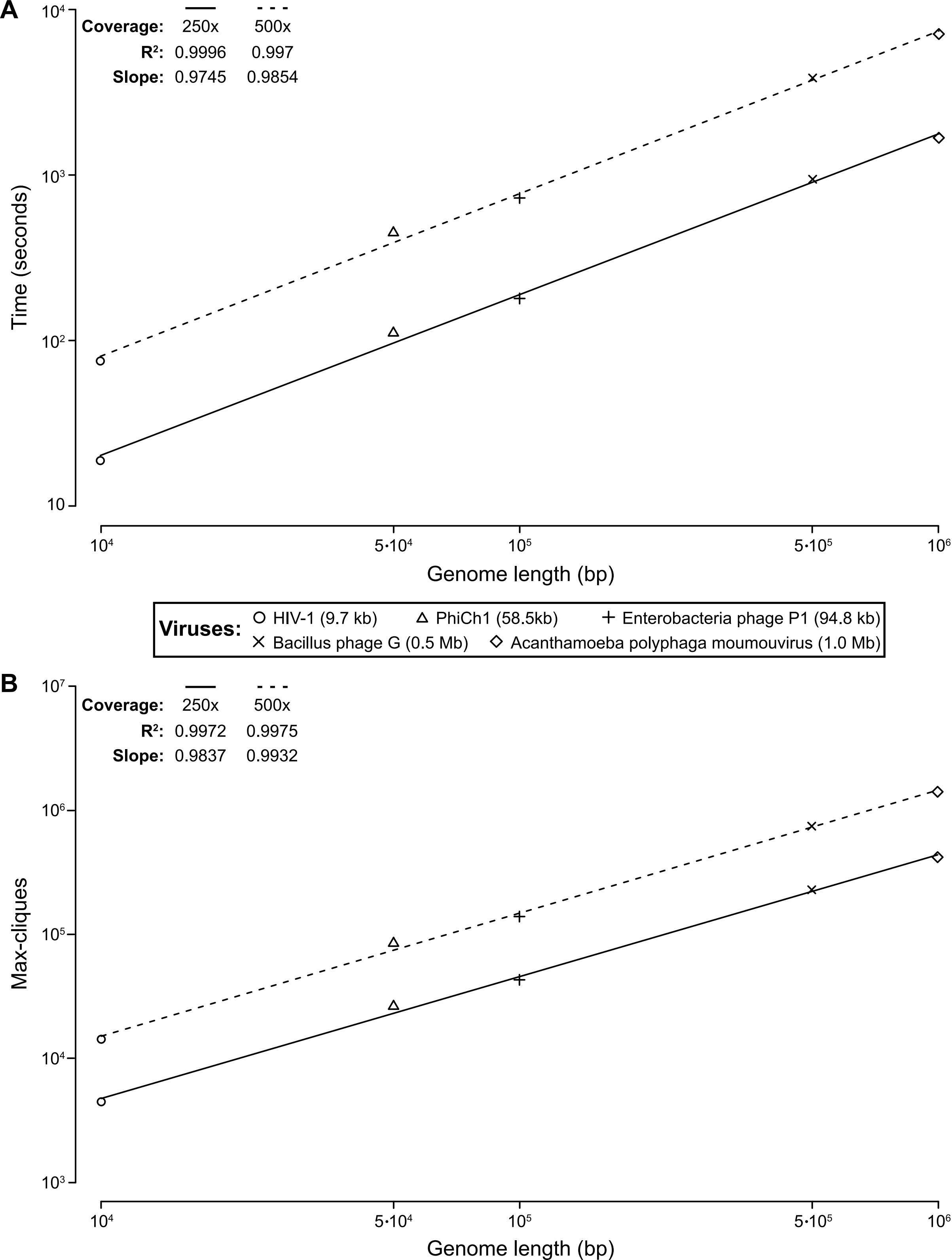

A
Coverage: 250x 500x
R²: 0.9996 0.997
Slope: 0.9745 0.9854
Time (seconds)
Genome length (bp)
Viruses:
HIV-1 (9.7 kb)
PhiCh1 (58.5kb)
Enterobacteria phage P1 (94.8 kb)
Bacillus phage G (0.5 Mb)
Acanthamoeba polyphaga moumouvirus (1.0 Mb)
B
Coverage: 250x 500x
R²: 0.9972 0.9975
Slope: 0.9837 0.9932
Max-cliques
Genome length (bp)
