# Supplementary material for: Viral Quasispecies Assembly via Maximal Clique Enumeration
Source: PLoS Comput Biol. 2014 Mar 27;10(3):e1003515. doi: 10.1371/journal.pcbi.1003515 (PMC3967922; doi:10.1371/journal.pcbi.1003515)
Supplement: Figure S6 — Edge definitions. Illustration of the compatible gaps condition of the sequence similarity criterion. Two reads and are aligned against the reference (left). This induces a direct read-to-read alignment of and (right). Case (1): No gaps in the reference alignments lead to a gapless read-to-read alignment, which renders the pair of reads an edge candidate. Case (2): Gaps in the reference alignment become eliminated in the direct read-to-read alignment implying an edge candidate. Case (3): The reference alignment leads to aligning –‘C’ against ‘C-’, which we interpret as aligning C against C, that is, virtually case (2) is in effect. Case (4): Gaps in the reference alignment that lead to gaps in the read-to-read alignment exclude the possibility of edges. Case (5): Similar to (3), but we interpret ‘-A’ against ‘C-’ as gap implying that no edge is possible. (PDF) [file pcbi.1003515.s006.pdf]

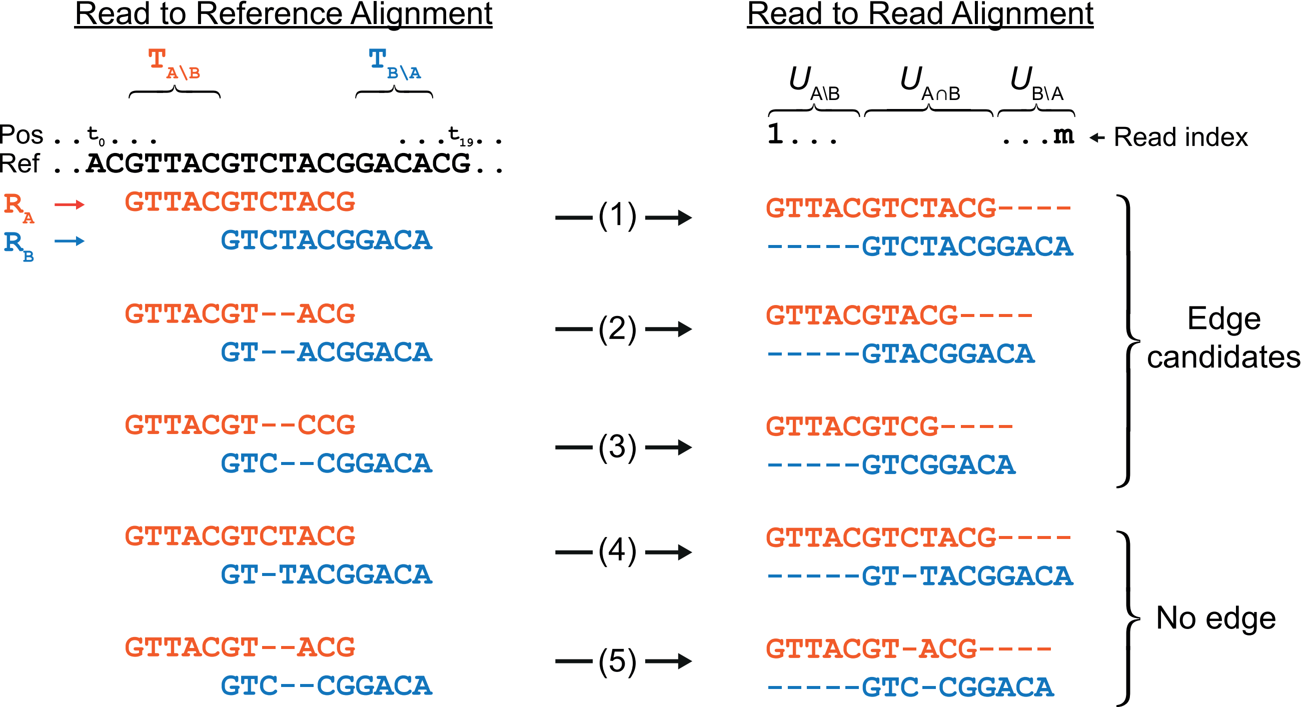

Read to Reference Alignment
Read to Read Alignment
T A\B
T B\A
U A\B
U A∩B
U B\A
Pos . . t0 . . . . t19 . .
Ref . . ACGTTACGTCTACGGACACG . .
1 . . . . . . m
Read index
R A
R B
GTTACGTCTACG
GTCTACGGACA
(1)
GTTACGTCTACG----
-----GTCTACGGACA
GTTACGT--ACG
GT--ACGGACA
(2)
GTTACGTACG----
-----GTACGGACA
GTTACGT--CCG
GTC--CGGACA
(3)
GTTACGTCG----
-----GTCGGACA
GTTACGTCTACG
GT-TACGGACA
(4)
GTTACGTCTACG----
-----GT-TACGGACA
GTTACGT--ACG
GTC--CGGACA
(5)
GTTACGT-ACG----
-----GTC-CGGACA
Edge candidates
No edge
